# Perceptions of Oral Health and Quality of Life among Parents in Kuwait

**DOI:** 10.3290/j.ohpd.b4043017

**Published:** 2023-04-24

**Authors:** Huda Nazar, Maddi Shyama, Jitendra Ariga

**Affiliations:** a Consultant Dental Public Health, Head Research and Survey Division, Dental Administration, Ministry of Health, Kuwait. Study design, planning, and conduction, supervision and administration, data collection, analysis and interpretation, wrote, revised, reviewed and edited the manuscript.; b Research and Survey Division, Dental Administration, Ministry of Health, Kuwait. Data analysis and interpretation, wrote, revised, reviewed and edited the manuscript.; c Director, School Oral Health Programme, Kuwait-Forsyth, Kuwait. Revised, reviewed and edited the manuscript.

**Keywords:** general health perceptions, Kuwait, oral health perceptions, parents, quality of life

## Abstract

**Purpose:**

To determine the self-perceived oral health and general health as well as the oral health-related quality of life (OHRQoL) among parents in Kuwait.

**Materials and Methods:**

This cross-sectional study was conducted among parents visiting the School Oral Health Programme (SOHP) clinics in all the governorates in Kuwait. Being a parent and able to read and understand the Arabic language was the inclusion criterion. A convenience sample of parents (n = 2357) were enrolled in this study, which was conducted using a self-administered questionnaire in Arabic. The questionnaire included previously validated questions and also questions designed for this study. The parents completed and returned the questionnaire while waiting for their children in the waiting area of the SOHP clinic.

**Results:**

The mean age of the parents was 38.3 ± 7.3 years. The majority (75.2%) of the participants perceived their oral health ‘excellent’, ‘very good’ or ‘good’ and 76.4% also rated their general health as ‘excellent’, ‘very good’ or ‘good’. Overall, higher levels of perceived oral health were reported more frequently by younger participants, females, Kuwaitis, and those who had higher levels of education (p < 0.001). Most of the participants (72.3%) were satisfied with their oral health, (91.0%) enjoyed eating their food and (79.0%) liked their smile. Perceived difficulty in chewing food was stated by only 21.0%, and very few (5.0%) had speech difficulties. Almost half (45.0%) mentioned that they had never had any dental and/or gum problems that affected their daily activities during the past 6 months, nor did such problems influence their social activities. Nearly two-thirds (61.0%) stated that they never had any difficulty in conversation, and half (49.0%) did not report any disturbance in their sleep. Cronbach’s alpha (0.89) showed a high degree of internal consistency between different OHRQoL responses.

**Conclusion:**

Most of the parents were satisfied with their oral health, which had an impact on their quality of life.

Oral health is an integral element of general health and well-being, contributing to the overall health-related quality of life for adults.^28,53^ Clinical indicators alone are no longer deemed adequate for determining the health status of individuals, since clinical measures alone do not consider subjective experience.^24^ Conceptualising and evaluating oral health in several dimensions confirm it as an important measure of general health.^25^

The self-perceived health of individuals is a crucial measure of health status, including both physical and psychological dimensions, and has been recognised as an essential predictor of health care.^18^ Self-rated oral health is an important indicator of overall oral health status, which has an influence on the comfort and quality of life of adults.^32,57^ Furthermore, the self-assessment of oral health has an important role in defining individuals’ habits concerning their oral health practices.^31^

Subjective indicators of oral health status are measures of the functional, social and psychological impact of oral disorders according to an individual’s experience, practice and behaviour with regard to disease.^35^ Measuring the subjective perceptions and perceived needs for oral health care in adults is important and can deliver essential evidence in formulating policies.^7^

Oral health-related quality of life (OHRQoL) is regarded as an essential section of the WHO’s Global Oral Health Programme, and is an important factor in the quality of life, influencing the condition, functional and emotional well-being of adults.^46,52^ The OHRQoL is an important subjective measure linked to oral health,^26^ and results from the interaction between oral health conditions, general health, and social as well as environmental factors.^37^ OHRQoL is a multidimensional construct that reflects individuals’ satisfaction with their oral health^52^ and includes the functional, social and psychological impacts of oral disease.^25^

Only a small number of OHRQoL studies have been conducted in the Middle-East countries, e.g. Saudi Arabia,^6,14,33,38,50^ Jordan,^21^ Syria and Egypt.^38^ Very limited information is available on oral health perceptions and OHRQoL among adults in Kuwait. In an earlier study in Kuwait, a majority of adults reported multiple oral health problems, and less than 10.0% reported no perceived oral health problems.^8^

In a previous study, the long-term effects of exposure to the School Oral Health Programme (SOHP) on OHRQoL of Kuwait University students was evaluated.^9^ The SOHP visits had a positive impact on the participants’ OHRQoL regarding their daily activities.^9^ In another study, the relationship between oral health knowledge, attitude and practices of primary school teachers and their OHRQoL was measured.^10^ Weak but statistically significant correlations were found between OHRQoL and knowledge, attitude and practice components. Self-esteem was the most commonly affected OHRQoL domain among the teachers.^10^ In another study, the OHRQoL among the parents and teachers of special-needs schoolchildren in Kuwait was assessed, in which oral health had a weak impact on the quality of life.^51^

Parents have an immeasurable impact on the psychological, emotional and intellectual well-being of their children.^34^ Adequate knowledge and attitude about oral health among parents initiates good oral habits in children.^54^ Habits and behaviours learned from the parents impacts oral health-related habits and thus the quality of life of children.^3,29^

The SOHP of Kuwait, established in 1983, is a comprehensive oral health programme which provides oral health education, preventive care and treatment for schoolchildren between the ages of 5 and 15 years in all the governorates of Kuwait.^13^ Parents and caregivers usually accompany their children to SOHP clinics to receive oral health care, i.e. preventive and restorative treatment.

As the perception of quality of life has a subjective component and can differ from one culture to another,^25^ obtaining information from Kuwait is very important. Data is lacking on the perceptions of oral health and OHRQoL among parents and caregivers in Kuwait. Hence, obtaining primary information could provide important evidence for policy makers in the planning and evaluation of oral health-care services. The objective of this study was to determine the perceptions of oral health and general health as well as OHRQoL among the parents visiting the SOHP in Kuwait.

## MATERIALS AND METHODS

This cross-sectional study was conducted among the parents visiting the SOHP clinics of Kuwait. The study protocol was approved by the Research Ethics Committee of SOHP, Kuwait-Forsyth. This study was conducted in accordance with the laws of the State of Kuwait, rules and regulations of the Ministry of Health, and was in full accordance with the World Medical Association Declaration in Helsinki. The study was conducted in all the governorates in Kuwait. Altogether, there were 2357 participants among the six governorates (Hawally, Farwaniya, Ahmadi, Al-Asima, Jahra, and Mubarak-Al-Kabir) (Table 1). The participants were the parents who accompanied their children to the SOHP center-based clinics to receive oral health care.

**Table 1:** Sociodemographic characteristics of the participants (n = 2357)

Variables	n (%)
**Age group (years)**	
20–29	257 (10.9)
30–39	1081 (45.9)
40–49	849 (36.0)
50–59	170 (7.2)
**Gender**	
Male	1226 (52.0)
Female	1131 (48.0)
**Nationality**	
Kuwaiti	1980 (84.0)
Non–Kuwaiti	377 (16.0)
**Marital status**	
Married	2154 (91.4)
Divorced/widowed	203 (8.6)
**Place of residence**	
Hawally	424 (18.0)
Farwaniya	582 (24.7)
Ahmadi	474 (20.1)
Al–Asima	429 (18.2)
Jahra	304 (12.9)
Mubarak Al–Kabir	144 (6.1)
**Educational level**	
No education	30 (1.3)
Intermediate school or less	261 (11.1)
High school	478 (20.3)
Diploma	516 (21.9)
University	925 (39.2)
University and above	147 (6.2)
**Medical history**	
Healthy	1942 (82.4)
Has medical condition	415 (17.6)
**Medical insurance**	
No insurance	1784 (75.7)
Has insurance	573 (24.3)


This study was conducted using a self-administered questionnaire in Arabic. The questionnaire was translated from English to Arabic and then back-translated into Arabic. The questionnaire was pretested in a pilot study with a comparable group of parents. The questionnaire included both previously validated questions^4,9,10,36,56^ and questions designed for this study. The questions were closed-ended. Informed consent was obtained from all participants. Being a parent and able to read and understand Arabic was the inclusion criterion. The study excluded older siblings accompanying their younger siblings. The questionnaires were distributed to the parents during morning and afternoon hours in the SOHP waiting area by well-trained dental hygienists and assistants. While waiting for their children in the waiting area, the parents completed the questionnaire. The questionnaires were assessed on site for completeness by the team, and the participants were asked to add any missing or incomplete information. A convenience sample of parents (n = 2357) participated in this study.

Independent variables included sociodemographics such as age, gender, nationality, area of residence, employment location (private or government), educational level, and marital status. The medical history was taken along with whether or not the parents had medical insurance. Age was categorised into four groups: 20–29 years, 30–39 years, 40–49 years, and 50–59 years.

The self-perceived oral health of the parents was evaluated by the Global Oral Health Indicator. The parents completed the global single question on perceived oral health (POH) (‘How would you rate the health of your teeth and mouth?’), with responses being excellent, very good, good, fair and poor.^4,36,56^ The perceived general health (PGH) (‘How would you rate your general health status?’) included a question about how the participant assesses general health, with responses being excellent, very good, good, fair, and poor.^3^

The section on subjective oral health status indicators included three questions on perceived oral health satisfaction. It was determined using the following questions: ‘Are you satisfied with your teeth/mouth?’, ‘Are you satisfied with your smile?’, and ‘Are you satisfied while eating food?’. All responses were recorded as ‘yes’ or ‘no’. The section on perceived difficulties comprised 2 questions: ‘Do you perceive difficulty in chewing food’ and ‘Do you perceive difficulty with speech?’. The responses were also recorded as ‘yes’ or ‘no’.

The OHRQoL questions consisted of three domains of physical, social and psychological (self-esteem) impairments, and one domain on sleep deprivation. The responses were scored on a scale from 1 to 4 for each item, with 1 meaning ‘all of the time – always’ and 4 meaning ‘none of the time – never’. For each of the questions, the participants were asked how frequently they had experienced the impact in the preceding six months.^9,10^

Parents also answered questions on their dental visits. Parents were further asked if they had had oral pain/ toothache within the preceding 6 months, with the response recorded as ‘yes’ or ‘no’.

### Statistical Analysis

The data were entered and analysed using Epi-Info 3.5.3 and SPSS for Windows version 24.0 (IBM; Armonk, NY, USA). Frequency distributions for all the variables were generated. The Χ^2^ test was used to assess the differences in percentage responses of the parents. Perceived oral and general health status, subjective oral health status and OHRQoL questions were compared by age, gender, nationality, place of residence, education level, marital status and medical history. Differences in the mean OHRQoL scores were evaluated using the t-test and ANOVA. Pearson’s correlation coefficient (r) was used to measure the inter-item and item-scale correlations among the participants. The internal consistency was measured by Cronbach’s alpha. Statistical significance was set at p < 0.05.

## RESULTS

Of 2590 questionnaires distributed to the participants, 2357 were complete, resulting in a 91.0% response rate. Overall, the survey sample comprised of 2357 adults (parents, guardians and caregivers). The mean age (±SD) of the participants was 38.3 ± 7.3 years. Around 46.0% of the participants were between 30 and 39 years old, and more than one-third (36.0%) were between 40 and 49 years of age. The gender distribution was 52.0% males and 48.0% females. Most of the participants were Kuwaitis (84.0%). More than one-third (39.2%) had either a college or a university qualification. A quarter (25.0%) resided in Farwaniya governorate. The majority of the participants (82.4%) were healthy and had no medical condition. Almost a quarter (24.2%) had medical insurance. Table 1 summarises the sociodemographic characteristics of the participants.

With regard to self-reported oral health, three-quarters (75.2%) of the participants perceived their oral health as ‘excellent’, ‘very good’ or ‘good’, 20.3% as ‘fair’ and only 4.5% indicated their oral health as ‘poor’. In addition, the majority (76.4%) of the participants reported their perceived general health as ‘excellent’, ‘very good’ or ‘good’, 14.6% stated it as ‘fair’, and less than 10.0% as ‘poor’.

Overall, higher levels of perceived oral health (excellent, very good and good) were reported more frequently by young participants, females, Kuwaitis and those who had higher levels of education (p < 0.001). The associations of these responses with the medical condition of the participants was not statistically significant (p = 0.832) (Table 2). Responses of the participants to the perceived general health question were very similar to their responses to the perceived oral health questions. Overall, higher levels of perceived general health (‘excellent’ and ‘very good’) were reported more frequently by young participants (p < 0.001), females (p = 0.024), Kuwaitis (p < 0.001), those who had higher levels of education (p = 0.007) and those who were healthy (did not have any medical condition) (p < 0.001) (Table 3).

**Table 2:** Distribution of perceived oral health of the participants according to different variables

Variables	Perceived oral health					p-value
	Excellent	Very good	Good	Fair	Poor	
**Age group (years)**						
20–29	36 (14.0)	102 (39.7)	65 (25.3)	43 (16.7)	11 (4.3)	
30–39	82 (7.6)	372 (34.4)	377 (34.9)	208 (19.2)	42 (3.9)	
40–49	67 (7.9)	245 (28.9)	306 (36.0)	186 (21.9)	45 (5.3)	
50–59	20 (11.8)	38 (22.4)	63 (37.1)	41 (24.1)	8 (4.7)	< 0.001
**Gender**						
Male	107 (8.7)	346 (28.2)	451 (36.8)	260 (21.2)	62 (5.1)	
Female	98 (8.7)	411 (36.3)	360 (31.8)	218 (19.3)	44 (3.9)	< 0.001
**Nationality**						
Kuwaiti	185 (9.3)	668 (33.7)	650 (32.8)	381 (19.2)	96 (4.9)	
Non–Kuwaiti	20 (5.3)	89 (23.6)	161 (42.7)	97 (25.7)	10 (2.7)	< 0.001
**Educational level**						
No education	2 (6.7)	7 (23.3)	12 (40.0)	7 (23.3)	2 (6.7)	
Intermediate school or less	33 (12.6)	55 (21.1)	83 (31.8)	69 (26.4)	21 (8.0)	
High school	28 (5.9)	143 (29.9)	167 (34.9)	115 (24.1)	25 (5.2)	
Diploma	44 (8.5)	186 (36.0)	171 (33.1)	98 (19.0)	17 (3.3)	
University	86 (9.3)	309 (33.4)	335 (36.2)	164 (17.7)	31 (3.4)	
Postgraduate	12 (8.2)	57 (38.8)	43 (29.3)	25 (17.0)	10 (6.8)	< 0.001
**Medical history**						
Healthy	171 (8.8)	632 (32.5)	660 (34.0)	393 (20.2)	86 (4.4)	
Has medical condition	34 (8.2)	125 (30.1)	151 (36.4)	85 (20.5)	20 (4.8)	0.832

Statistical significance set at p < 0.05.

**Table 3:** Distribution of perceived general health of the participants according to different variables

Variables	Perceived general health					p-value
	Excellent	Very good	Good	Fair	Poor	
**Age group (years)**						
20–29	20 (7.8)	107 (41.6)	83 (32.3)	23 (8.9)	24 (9.3)	
30–39	52 (4.8)	371 (34.3)	373 (34.5)	176 (16.3)	109 (10.1)	
40–49	36 (4.2)	268 (31.6)	354 (41.7)	118 (13.9)	73 (8.6)	
50–59	5 (2.9)	52 (30.6)	73 (42.9)	27 (15.9)	13 (7.6)	< 0.001
**Gender**						
Male	60 (4.9)	381 (31.1)	468 (38.2)	199 (16.2)	118 (9.6)	
Female	53 (4.7)	417 (36.9)	415 (36.7)	145 (12.8)	101 (8.9)	0.024
**Nationality**						
Kuwaiti	98 (4.9)	698 (35.3)	709 (35.8)	274 (13.8)	201 (10.2)	
Non–Kuwaiti	14 (4.0)	100 (26.5)	174 (46.2)	70 (18.6)	18 (4.8)	< 0.001
**Educational level**						
No education	2 (6.7)	9 (30.0)	11 (36.7)	4 (13.3)	4 (13.3)	
Intermediate school or less	6 (2.3)	84 (32.2)	93 (35.6)	39 (14.9)	39 (14.9)	
High school	25 (5.2)	137 (28.7)	187 (39.1)	84 (17.6)	45 (9.4)	
Diploma	31 (6.0)	185 (35.9)	183 (35.5)	77 (14.9)	40 (7.8)	
University	41 (4.4)	325 (35.1)	367 (39.7)	121 (13.1)	71 (7.7)	
Postgraduate	8 (5.4)	58 (39.5)	42 (28.6)	19 (12.9)	20 (13.6)	0.007
**Medical history**						
Healthy	99 (5.1)	709 (36.5)	660 (34.0)	278 (14.3)	196 (10.1)	
Has medical condition	14 (3.4)	89 (21.4)	223 (53.7)	66 (15.9)	23 (5.5)	< 0.001

Statistical significance set at p < 0.05.

The higher levels of perceived oral health (‘excellent’, ‘very good’ and ‘good’) were statistically significantly associated with higher levels of perceived general health (p < 0.001). The lowest level of perceived oral health (‘poor’) reported by the participants was associated with lowest level of perceived general health. Pearson’s correlation coefficient was statistically significant (r = 0.460; p < 0.001) (Table 4).

**Table 4:** Correlations between responses to perceived oral health and general health (POH and PGH) questions among the participants

	PGH					p-value (χ2)	Pearson correlation
	Excellent	Very good	Good	Fair	Poor		
**POH**							
Excellent	24 (11.7)	123 (60.0)	45 (22.0)	9 (4.4)	4 (2.0)		
Very good	55 (7.3)	403 (53.2)	234 (30.9)	50 (6.6)	15 (2.0)		
Good	23 (2.8)	182 (22.4)	398 (49.1)	155 (19.1)	53 (6.5)		
Fair	9 (1.9)	79 (16.5)	189 (39.5)	106 (22.2)	95 (19.9)		
Poor	2 (1.9)	11 (10.4)	17 (16.0)	24 (22.6)	52 (49.1)	< 0.001	0.464, p < 0.001

Statistical significance set at p < 0.05.

Satisfaction with their oral health (mouth and teeth) was stated by 72.3% of the participants. The majority (91.0%) specified their satisfaction and enjoyed eating their food. More than three-quarters of the participants indicated that they liked their smile (79.0%). Perceived difficulty in chewing food was stated by only 21.0%. Very few, only 5.0%, felt that they had speech difficulties (Table 5). Perceived difficulty in chewing food among the participants was statistically significantly associated with speech difficulties. Participants who had difficulties in chewing their food were more likely to have experienced pronunciation or speech difficulties (OR=5.4; 95% CI=3.7-7.9). About 13.5% of the participants who perceived difficulty in chewing their food had speech difficulties, compared to only 3.0% of participants reporting speech difficulty without difficulty in chewing their food (p < 0.001).

**Table 5:** Perceived subjective measures of oral health among the participants

Variable	n	%
**Perceived satisfaction with mouth and teeth**		
Yes	1705	72.3
No	652	27.7
**Perceived satisfaction while eating food**		
Yes	2138	90.7
No	219	9.3
**Perceived satisfaction with smile**		
Yes	1861	79.0
No	496	21.0
**Perceived difficulty in chewing food**		
No	1869	79.3
Yes	488	20.7
**Perceived difficulty with speech**		
No	2239	95.0
Yes	118	5.0


The participants’ OHRQoL responses are shown in Table 6. Almost half of the participants (45.0%), mentioned that they never had any teeth and/or gum problems that affected their daily activities during the past 6 months. Moreover, around half of the participants (45.0%) were not affected by such problems in their social life/activities or self-esteem. Nearly two-thirds (61.0%) mentioned that they never had any difficulty in conversation. Half of the participants (49.0%) did not experience any disturbance in their sleep.

**Table 6:** OHRQoL responses of the participants during the past six months

Variables	n	%
**How often have problems with your teeth or gums during the past six months affected your daily activities?**		
All of the time	269	11.4
Most of the time	368	15.6
Some of the time	660	28.0
Never	1060	45.0
**How often have problems with your teeth or gums during the past six months affected your social activities?**		
All of the time	330	14.0
Most of the time	365	15.5
Some of the time	601	25.5
Never	1061	45.0
**How often have problems with your teeth or gums during the past six months caused you to avoid conversations?**		
All of the time	340	14.4
Most of the time	217	9.2
Some of the time	364	15.4
Never	1436	60.9
**How often have problems with your teeth or gums during the past six months disturbed your sleep?**		
All of the time	283	12.0
Most of the time	255	10.8
Some of the time	669	28.4
Never	1150	48.8


The mean OHRQoL score was 12.5 ± 3.7. OHRQoL scores were statistically significantly higher (13.2 ± 3.6) in the youngest age group (20–29 years) compared to participants over 30 years of age (12.5 ± 3.6; p < 0.001). Females had slightly higher OHRQoL (12.5 ± 3.6) than did males (12.4 ± 3.7; p = 0.041). There were also differences in OHRQoL among the governorates. Participants residing in Al-Asima governorate had a statistically significantly higher OHRQoL score 14.3 ± 2.0 than did those residing in Farwaniya governorate (7.9 ± 3.6; p < 0.001). OHRQoL was statistically significantly greater among participants with a university education (12.9 ± 3.7) vs those with no education (11.2 ± 4.0; p < 0.001). Participants who were married and had no medical condition had a lower OHRQoL (Table 7).

**Table 7:** Mean (±SD) OHRQoL of participants according to sociodemographic variables

Variables	n	%	OHRQoL	p-value
**Age group (years)**				
20–29	257	10.9	13.2 ± 3.6	
30–39	1081	45.9	12.1 ± 3.8	
40–49	849	36.0	12.6 ± 3.5	
50–59	170	7.2	12.8 ± 3.4	< 0.001
**Gender**				
Male	1226	52.0	12.4 ± 3.7	
Female	1131	48.0	12.5 ± 3.6	0.041
**Nationality**				
Kuwaiti	1980	84.0	12.4 ± 3.7	
Non-Kuwaiti	377	16.0	12.5 ± 3.6	0.361
**Marital status**				
Married	2154	91.4	12.4 ± 3.7	
Divorced/widowed	203	8.6	12.9 ± 3.5	0.016
**Place of residence**				
Hawally	424	18.0	14.1 ± 2.2	
Farwaniya	582	24.7	7.9 ± 3.6	
Ahmadi	474	20.1	13.9 ± 2.2	
Al-Asima	429	18.2	14.3 ± 2.0	
Jahra	304	12.9	13.3 ± 2.3	
Mubarak Al–Kabir	144	6.1	13.9 ± 2.2	< 0.001
**Educational level**				
No education	30	1.3	11.2 ± 4.0	
Intermediate school or less	261	11.1	12.1 ± 3.6	
High school	478	20.3	11.7 ± 3.7	
Diploma	516	21.9	12.6 ± 3.5	
University	925	39.2	12.9 ± 3.7	
University and above	147	6.2	12.4 ± 4.3	< 0.001
**Medical history**				
Healthy	1942	82.4	12.4 ± 3.8	
Has medical condition	415	17.6	12.7 ± 3.4	0.001

Statistical significance set at p < 0.05.

All the inter-item correlations were positive. The strongest inter-item correlations were observed between ‘disturbed sleep’ and ‘caused avoidance of conversation’ (r = 0.77). Further, strong inter-item correlations were seen between ‘caused avoidance of conversation’ and ‘affected daily activities’ (r = 0.69); ‘disturbed sleep’ and ‘affected daily activities’ (r = 0.68); ‘affected social activities’ and ‘affected daily activities’ (r = 0.67) (Table 8). Cronbach’s alpha (0.89) showed a high degree of internal consistency between different OHRQoL responses.

**Table 8:** Correlations (r) of OHRQoL responses among the participants

Variables	Affected daily activities	Affected social activities	Caused avoidance of conversations	Disturbed sleep
Affected daily activities	1.00			
Affected social activities	0.67*	1.00		
Caused avoidance of conversations	0.69*	0.65*	1.00	
Caused disturbance in sleep	0.68*	0.60*	0.77*	1.00

*Correlation is significant at the 0.01 level

Almost one-third of adults (31.0%) reported that they had visited a dentist more than a year ago while, 26.0% had dental visits in the past 12 months and about 41.5% less than six months ago. Regarding the frequency of dental visits among the age groups, nearly half (47.5%) of the participants in the 20- to 29-year age group had visited a dentist less than six months ago, while more than one-third (37.0%) of 50- to 59-year age group had done so (p = 0.019). Nearly half of females (48.6%) had visited a dentist less than six months ago, wherease about one-third of the males did (35.0%; p < 0.001). More Kuwaitis (43.0%) reported dental care visits in the last six months than did non-Kuwaitis (36.0%; p = 0.001).

Nearly one-third (32.0%) of the participants had experienced or perceived oral pain within the past six months. Oral pain was more prevalent among the participants residing in Jahra governorate (42.1%)than among those in Al-Asima (27.3%; p < 0.001). Oral pain was more prevalent among those who had a medical condition (40.0%) than among those who were healthy (30.0%; p < 0.001). Oral pain was associated with perceived difficulty in chewing food among the participants. Participants who experienced difficulties in chewing their food were more likely to have experienced oral pain/toothache (OR=3.1; 95% CI=2.5-3.8). More than half (52.3%) of the participants with poor chewing ability had oral pain, vs 26.3% of participants who had no difficulty in chewing their food (p < 0.001).

## DISCUSSION

This study was conducted to determine the self-rated oral and general health (perceptions of oral and general health), subjective oral health status, OHRQoL, dental visits, and occurrence of oral pain among parents visiting the SOHP clinics in all governorates of Kuwait. A high response rate in this study (91.0%) showed a high degree of interest among the participants regarding their oral health.

This is the first study on the association between POH and PGH conducted in Kuwait among parents, including the relationship of POH and PGH with some sociodemographic variables. In the present study, a large proportion of the study participants (75.2%) viewed their POH positively, rating their POH as ‘excellent’, ‘very good’ or ‘good’. This high proportion might be attributed to factors such as good knowledge and practice of oral health among parents. This overall proportion of POH is higher than that reported for other populations. Only up to half of Yemeni adults (51%) rated their POH as ‘good’, ‘very good’ or ‘excellent’,^4^ and 58.3% of adult Nigerians rated their oral health status as ‘very good’ or ‘good’.^41^

In this study, the proportion of self-rated oral health is similar to and in agreement with other studies conducted in various populations, e.g. Saudia Arabia (75.1%),^33^ South Africa (76.3%)^42^ and Brazil (74.4%),^12^ but lower than that reported in Australia (83.0%).^39^ Overall, only a quarter (25.0%) of the participants rated their oral health status as ‘fair’ or ‘poor’. Compared to this study, higher proportions of adults in Yemen (49.0%),^4^ and Nigeria (36.4%)^41^ viewed their oral health status as ‘fair’ or ‘poor’. Few participants (4.5%) in this study reported having poor self-rated oral health, which is lower than reported in a study among Canadian adults.^30^

In this study, parents had very good knowledge of oral health. However, in a previous study in Kuwait, the level of dental knowledge was higher among teachers than mothers.^45^ Hence, group presentations and one-to-one communications at school were recommended to improve the positive attitudes and dental health behaviour among mothers.^45^ Also, the oral health knowledge among mothers in Saudi Arabia was uncertain, while their attitudes towards prevention were positive.^11^

In a recent study, the establishment of school-based oral health programmes with active involvement of parents and teachers was considered important to promote healthy lifestyles and improve the oral health among schoolchildren in Palestine.^2^ Similarly, a study in Jordan indicated the need for the establishment of school-based oral health programmes to influence the oral health behaviour of children and parents.^48^ The SOHP of Kuwait is a comprehensive school-based oral health programme which follows the WHO’s Health Promoting School concept.^13^

In this study, higher levels of perceived oral and general health were reported more frequently by younger participants. In agreement with an earlier study among Yemeni adults,^4^ younger Kuwaiti participants in this study rated their oral and general health more positively than did the older age groups, and indicated better self-reported health than did participants in previous studies in Greece and Brazil.^23,55^ Older individuals may be unable to maintain good oral hygiene, and in comparison to young adults, could experience age-related health problems that decrease the ability to resist diseases. Also, age may be a relevant factor, as several diseases are more prevalent among older individuals, and self-evaluation of health frequently deteriorates with age.^4,55^

Educational level was an important determinant of self-rated oral and general health. Participants with higher educational levels perceived their oral and general health as being better than did participants with lower educational levels. This was similar in previous studies among adults in Yemen,^4^ South Africa,^42^ Greece^23^ and Brazil.^55^ Conversely, among adults in Saudi Arabia, there was no association between education and their perceived self-rated oral health status.^33^ Individuals who are well educated experience better self-reported health, while less education is related to self-reported poor health.^47^ Education may result in increased understanding of the importance of preventive oral health care, as better-educated individuals have more knowledge and awareness of their oral health.^4^

In the current study, gender played a role in terms of perceived health: more females than males reported better oral and general health. Females usually take more interest in their oral and general health and appearance, which may be reflected in their better preventive oral health-care practices and self-rated response.^27,30^ In contrast, in previous studies, females reported worse self-rated health.^4,23,55^ However, self-rated health was not associated with gender in Irish adults.^22^ Overall, in the present study, there was a statistically significant association and correlation between the POH and PGH among the participants, concurring with other studies which showed a statistically significant relationship between the POH and PGH.^4,30,49^ The oral cavity may be one of the first sites at which several systemic diseases, many infections, inflammatory diseases and nutritional deficiencies become apparent.^15,20,49^

The majority of the participants (72.3%) in this study expressed satisfaction with their oral health. Similarly, in an different study among students at Kuwait University, most of them (60.0%) were satisfied with their oral health and the appearance of their teeth.^5^ Conversely, more than half (59.0%) of adults in Brazil reported being dissatisfied with their mouth and teeth.^19^ Overall, discomfort when chewing food was expressed by less than 25% of the participants, which was in agreement with a recent study, in which feeling uncomfortable eating food was stated by 22.0% of indigenous adults in Canada.^30^ This frequency was lower than that reported in a previous study, in which, one-third (33.5%) of Brazilian adults stated that eating was the most frequently impacted daily performance and had difficulty while eating.^19^ However, in other studies, only 16.0% of Thai adults and Canadian adults expressed discomfort when chewing.^30,58^

In this study, the majority were satisfied with their smile. Only a small number of participants expressed difficulty in speech. Conversely, in previous studies, smiling/speaking were the most commonly affected items in the domain of impact on daily performance (27.3%) among Brazilian adults.^1,19^ The perceived difficulty in chewing was associated with speech difficulties. Participants who had limitations in their chewing or biting capacity also experienced speech difficulties. In an different study among Brazilian adults, nearly half (49.4%) reported their chewing ability to be ‘fair’, ‘poor’, or ‘very poor’, and more than one-third (37.4%) gave these ratings to speech ability.^44^

Almost half of the participants reported that their oral health (dental or gum problems) had never interrupted their daily activities, social activities or sleep, while less than 40.0% had difficulty conversing. Also, less than 15% reported ‘always’ having difficult conversing. The OHRQoL of the participants in this study resemble that of the previous study in Kuwait among university students, where only half of them stated that their activities and speech were affected by oral health problems.^9^

The OHRQoL among the participants in this study was statistically significantly better among the youngest age group, females, those residing in Al-Asima governorate, and those who had a higher education qualification. Similar to this study, in a previous study in Kuwait among school teachers, OHRQoL was better among females and those who had higher qualifications.^10^ Conversely, teachers residing in Al-Asima and Hawally governorates in Kuwait had the lowest of OHRQoL.^10^ Comparable to this study, in an earlier study in Kuwait, OHRQoL was higher among university-educated parents compared to those with primary education.^51^

In agreement with the present study, a higher educational level had a statistically significant positive impact on the quality of life of adults in Greece.^43^ However, in contrast to this study, a higher educational level was associated with poor OHRQoL among Brazilain adults.^16^ As opposed to the present study, females had a poorer OHRQoL when compared to males in Saudi Arabia.^6^ This study showed an excellent internal consistency between the different OHRQoL responses (0.89). This was comparable to the earlier study in Kuwait among parents and teachers, where Cronbach’s alpha for reliability and validity of OHRQoL was 0.83.^51^

In this study, 25.6% of the participants reported that they had visited a dentist during the last 12 months and about 41.5% less than six months ago. Compared to the present study, in an earlier study in Kuwait, a higher percentage of participants (39.0%) had visited a dentist during the previous 12 months.^17^ In a recent study among adult employees in Kuwait by Nazar et al,^40^ 55.0% of the adults reported that they had visited a dentist during the previous 12 months. In the present study, nearly one-third (31.7%) of the participants had experienced oral pain within the past six months. Similarly, in a recent study, 33.0% of adults had perceived oral pain at the time of their examination.^40^ In previous studies in Kuwait, more than two-thirds (69.0%) of adults^17^ and 70.0% of Kuwait University students^5^ stated that toothache was the main reason for their dental visits.

One limitation of the present study is that it was conducted on a convenience sample of adults, among the parents visiting SOHP in Kuwait, and thus may not be representative of adults in Kuwait. Furthermore, this study was based on a cross-sectional survey in only a selected group of adults. Hence, it is difficult to determine how various factors may influence the perceptions of oral and general health as well as OHRQoL.

## CONCLUSION

Most of the parents were satisfied with their oral health, which had an impact on their quality of life. Further studies should be planned to assess the various factors that may influence OHRQoL among a wider and more representative range of the population in Kuwait.

## References

[ref1] Abegg C, Fontanive VN, Tsakos G, Davoglio RS, de Oliveira MM (2015). Adapting and testing the oral impacts on daily performances among adults and elderly in Brazil. Gerodontol.

[ref2] Abuhaloob L, Petersen PE (2021). Oral health status and oral health behaviour among 5- to 6-year-old Palestinian schoolchildren –towards engagement of parents and schoolteachers for oral health through schools. Oral Health Prev Dent.

[ref3] Adair PM, Burnside G, Pine CM (2013). Analysis of health behaviour change interventions for preventing dental caries delivered in primary schools. Caries Res.

[ref4] Alhajj MN, Halboub E, Amran AG, Alkheraif AA, Al-Sanabani FA, Al-Makramani BM (2019). Link between perceived oral and general health status among Yemeni adult dental patients. BMC Oral Health.

[ref5] Al-Hussaini R, Al-Kandari M, Hamadi T, Al-Mutawa A, Honkala S, Memon A (2003). Dental health knowledge, attitudes and behaviour among students at the Kuwait university health sciences centre. Med Princ Pract.

[ref6] Alrumyyan A, Quwayhis S, Meaigel S, Almedlej R, Alolaiq R, Bin Nafesah R (2020). Oral health-related quality of life and oral hygiene practice of adults with fixed dental prostheses in Riyadh, Saudi Arabia. J Int Soc Prev Community Dent.

[ref7] AlShahrani I, Tikare S, Togoo RA, AlAsere YH, AlAsmari AA (2015). Self-perception of personal oral health in Saudi population: a social media approach. East Mediterr Health J.

[ref8] Al-Shammari KF, Al-Ansari JM, Al-Khabbaz AK, Dashti A, Honkala EJ (2007). Self-reported oral hygiene habits and oral health problems of Kuwaiti adults. Med Princ Pract.

[ref9] Alsumait A, ElSalhy M, Amin M (2015). Long-term effects of school-based oral health program on oral health knowledge and practices and oral health-related quality of life. Med Princ Pract.

[ref10] Alsumait A, ElSalhy M, Almunezaa E, Ariga J, Al-Mutawa S, Amin M (2016). Relationship between oral health knowledge, attitude and practices of primary school teachers and their oral health-related quality of life: A cross-sectional study. Oral Health Prev Dent.

[ref11] Al-Tamimi S, Petersen PE (1998). Oral health situation of schoolchildren, mothers and schoolteachers in Saudi Arabia. Int Dent J.

[ref12] Andrade FB, Lebrão ML, Santos JL, Duarte YA, Teixeira DS (2012). Factors related to poor self-perceived oral health among community-dwelling elderly individuals in São Paulo, Brazil. Cad Saude Publica.

[ref13] Ariga J, Al-Mutawa S, Nazar H (2014). School Oral Health Program in Kuwait. Med Princ Pract.

[ref14] Atieh MA (2008). Arabic version of the Geriatric oral health assessment index. Gerodontol.

[ref15] Babu NC, Gomes AJ (2011). Systemic manifestations of oral diseases. J Oral Maxillofac Pathol.

[ref16] Bado FMR, De Checchi MHR, Cortellazzi KL, Ju X, Jamieson L, Mialhe FL (2020). Oral health literacy, self-rated oral health, and oral health-related quality of life in Brazilian adults. Eur J Oral Sci.

[ref17] Behbehani JM, Shah NM (2002). Oral health in Kuwait before the Gulf War. Med Princ Pract.

[ref18] Bonner WIA, Weiler R, Orisatoki R, Lu X, Andkhoie M, Ramsay D (2017). Determinants of self-perceived health for Canadians aged 40 and older and policy implications. Int J Equity Health.

[ref19] Chalub LLFH, Ferreira RC, Vargas AMD (2017). Influence of functional dentition on satisfaction with oral health and impacts on daily performance among Brazilian adults: a population-based cross-sectional study. BMC Oral Health.

[ref20] Chang C-S, Chang FM, Nakagaki H, Morita I, Tsuboi S, Sakakibara Y (2010). Comparison of the oral health and self-rated general health status of undergraduate students in Taiwan and Japan. J Dent Sci.

[ref21] Daradkeh S, Khader YS (2008). Translation and validation of the Arabic version of the geriatric oral health assessment index (GOHAI). J Oral Sci.

[ref22] Darker CD, Donnelly-Swift E, Whiston L, Moore F, Barry JM (2016). Determinants of self-rated health in an Irish deprived suburban population –a cross sectional face-to-face household survey. BMC Public Health.

[ref23] Darviri C, Fouka G, Gnardellis C, Artemiadis AK, Tigani X, Alexopoulos EC (2012). Determinants of self-rated health in a representative sample of a rural population: a cross-sectional study in Greece. Int J Environ Res Public Health.

[ref24] Gerritsen AE, Allen PF, Witter DJ, Bronkhorst EM, Creugers NH (2010). Tooth loss and oral health-related quality of life: a systematic review and meta-analysis. Health Qual Life Outcomes.

[ref25] Gift HC, Atchison KA, Dayton CM (1997). Conceptualizing oral health and oral health-related quality of life. Soc Sci Med.

[ref26] Glick M, Williams DM, Kleinman DV, Vujicic M, Watt RG, Weyant RJ (2016). A new definition for oral health developed by the FDI World Dental Federation opens the door to a universal definition of oral health. Br Dent J.

[ref27] Hamasha AA, Alshehri A, Alshubaiki A, Alssafi F, Alamam H, Alshunaiber R (2018). Gender-specific oral health beliefs and behaviors among adult patients attending King Abdulaziz Medical City in Riyadh. Saudi Dent J.

[ref28] Hescot P (2017). The new definition of oral health and relationship between oral health and quality of life. Chin J Dent Res.

[ref29] Hooley M, Skouteris H, Boganin C, Satur J, Kilpatrick N (2012). Parental influence and the development of dental caries in children aged 0–6 years: a systematic review of the literature. J Dent.

[ref30] Hussain A, Jaimes SB, Crizzle AM (2021). Predictors of self-rated oral health in Canadian indigenous adults. BMC Oral Health.

[ref31] Kakudate N, Morita M, Fukuhara S, Sugai M, Nagayama M, Kawanami M (2010). Application of self-efficacy theory in dental clinical practice. Oral Dis.

[ref32] Kojima A, Ekuni D, Mizutani S, Furuta M, Irie K, Azuma T Relationships between self-rated oral health, subjective symptoms, oral health behavior and clinical conditions in Japanese university students: a cross-sectional survey at Okayama University. BMC Oral Health2013.

[ref33] Kotha SB, Chaudhary M, Terkawi S, Ahmed M, Ghabban SN, Fernandez RAA (2017). Correlation of perceived self-rated oral health status with various dental health and awareness factors. J Int Soc Prev Community Dent.

[ref34] Kratochwill TR, McDonald L, Levin JR, Scalia PA, Coover G (2009). Families and schools together: an experimental study of multi-family support groups for children at risk. J Sch Psychol.

[ref35] Locker D, Miller Y (1994). Evaluation of subjective oral health status indicators. J Public Health Dent.

[ref36] Locker D, Mscn EW, Jokovic A (2005). What do older adults’ global self-ratings of oral health measure?. J Public Health Dent.

[ref37] Locker D, Allen F (2007). What do measures of ‘oral health-related quality of life’ measure?. Community Dent Oral Epidemiol.

[ref38] McGrath C, Alkhatib MN, Al-Munif M, Bedi R, Zaki AS (2003). Translation and validation of an Arabic version of the UK oral health related quality of life measure (OHQoL-UK) in Syria, Egypt and Saudi Arabia. Community Dent Health.

[ref39] Mejia G, Armfield JM, Jamieson LM (2014). Self-rated oral health and oral health-related factors: the role of social inequality. Aust Dent J.

[ref40] Nazar H, Shyama M, Ariga J, Alsumait A (2021). Oral health status among adult employees in Kuwait. Oral Health Prev Dent.

[ref41] Olusile AO, Adeniyi AA, Orebanjo O (2014). Self-rated oral health status, oral health service utilization, and oral hygiene practices among adult Nigerians. BMC Oral Health.

[ref42] Olutola BG, Ayo-Yusuf OA (2012). Socio-environmental factors associated with self-rated oral health in South Africa: a multilevel effects model. Int J Environ Res Public Health.

[ref43] Papaioannou W, Oulis CJ, Latsou D, Yfantopoulos J (2011). Oral health-related quality of life of Greek adults: A cross-sectional study. Int J Dent.

[ref44] Pattussi MP, Peres KG, Boing AF, Peres MA, da Costa JS (2010). Self-rated oral health and associated factors in Brazilian elders. Community Dent Oral Epidemiol.

[ref45] Petersen PE, Hadi R, Al-Zaabi FS, Hussein JM, Behbehani JM, Skougaard MR, Vigild M (1990). Dental knowledge, attitudes, and behaviour among Kuwaiti mothers and schoolteachers. J Pedod.

[ref46] Petersen PE (2003). The World Oral Health Report 2003: continuous improvement of oral health in the 21st century –the approach of the WHO global oral health programme. Community Dent Oral Epidemiol.

[ref47] Raghupathi V, Raghupathi W (2020). The influence of education on health: an empirical assessment of OECD countries for the period 1995–2015. Arch Public Health.

[ref48] Rajab LD, Petersen PE, Bakeen G, Hamdan MA (2002). Oral health behaviour of schoolchildren and parents in Jordan. Int J Paediatr Dent.

[ref49] Reissmann DR, John MT, Schierz O, Kriston L, Hinz A (2013). Association between perceived oral and general health. J Dent.

[ref50] Shaikh H, Shilpa RH, Fatima A, Asawa K, Kannan K, Lankar A (2020). Assessment of oral health-related quality of life among expatriate working population, Saudi Arabia: A cross-sectional study. J Int Soc Prev Community Dent.

[ref51] Shyama M, Honkala S, Al-Mutawa SA, Honkala E (2013). Oral health-related quality of life among parents and teachers of disabled schoolchildren in Kuwait. Med Princ Pract.

[ref52] Sischo L, Broder HL (2011). Oral health-related quality of life: what, why, how, and future implications. J Dent Res.

[ref53] Spanemberg JC, Cardoso JA, Slob EMGB, López-López J (2019). Quality of life related to oral health and its impact in adults. J Stomatol Oral Maxillofac Surg.

[ref54] Szatko F, Wierzbicka M, Dybizbanska E, Struzycka I, Iwanicka-Frankowska E (2004). Oral health of Polish three-year-olds and mothers’ oral health-related knowledge. Community Dent Health.

[ref55] Szwarcwald CL, Souza-Júnior PR, Esteves MA, Damacena GN, Viacava F (2005). Socio-demographic determinants of self-rated health in Brazil. Cad Saude Publica.

[ref56] Thomson WM, Mejia GC, Broadbent JM, Poulton R (2012). Construct validity of Locker’s global oral health item. J Dent Res.

[ref57] Ueno M, Zaitsu T, Ohara S, Wright C, Kawaguchi Y (2015). Factors influencing perceived oral health of Japanese middle-aged adults. Asia Pac J Public Health.

[ref58] Yiengprugsawan V, Somkotra T, Seubsman SA, Sleigh AC, Thai Cohort Study Team (2011). Oral health-related quality of life among a large national cohort of 87,134 Thai adults. Health Qual Life Outcomes.

